# PLOD2 contributes to drug resistance in laryngeal cancer by promoting cancer stem cell-like characteristics

**DOI:** 10.1186/s12885-019-6029-y

**Published:** 2019-08-27

**Authors:** Xiaoli Sheng, Yunxian Li, Yixuan Li, Wenlin Liu, Zhongming Lu, Jiandong Zhan, Mimi Xu, Liangsi Chen, Xiaoning Luo, Gang Cai, Siyi Zhang

**Affiliations:** 1 Second School of Clinical Medicine, Southern Medical University, Guangzhou, Guangdong China; 2 Department of Otorhinolaryngology, Guangdong Provincial People’s Hospital & Guangdong Academy of Medical Sciences, No.106, Zhongshan Er Road, Guangzhou, 510080 Guangdong Province China; 3 Shantou University Medical College, Shantou, Guangdong China; 4 Head and Neck Surgery of Sun Yat-sen University Cancer Center, Guangzhou, Guangdong China; 5 Department of Otorhinolaryngology, The Sixth Affiliated Hospital of Guangzhou Medical University, Qingyuan People’s Hospital, Qingyuan, Guangdong China; 6 The Fifth Affiliated Hospital of Guangzhou Medical University, No.621, Gangwan Road, Guangzhou, 510700 Guangdong Province China

**Keywords:** Laryngeal squamous cell carcinoma, PLOD2, Drug resistance, Cancer stem cells, Wnt/β-catenin pathway

## Abstract

**Background:**

Advanced stage laryngeal squamous cell carcinoma (LSCC) presents a poor prognosis; thus, there is a great need to identify novel prognostic molecular markers. Procollagen-lysine, 2-oxoglutarate 5-dioxygenase 2 (PLOD2) is thought to be a novel prognostic factor in several cancers, but its role in LSCC remains unknown. Cancer stem cells (CSCs) are responsible for most instances of tumor recurrence and the development of drug resistance and have been proven to be present in head and neck cancers. Our preliminary study indicated that PLOD2 was elevated in LSCC tissues; therefore, we hypothesized that PLOD2 is related to the prognosis of LSCC patients and aimed to explore the role and underlying mechanism of PLOD2 in LSCC.

**Methods:**

We validated the prognostic role of PLOD2 in 114 LSCC patients by immunohistochemistry. Stable PLOD2-overexpressing Hep-2 and FaDu cells were established and assessed by molecular biology and biochemistry methods both in vitro and in vivo.

**Results:**

We confirmed that PLOD2 overexpression was correlated with poor prognosis in LSCC patients. PLOD2 overexpression strengthened the CSC-like properties of Hep-2 and FaDu cells, activated the Wnt signaling pathway and conferred drug resistance in LSCC in vitro and in vivo.

**Conclusions:**

We found that PLOD2 could serve as a prognostic marker in patients with LSCC and confer drug resistance in LSCC by increasing CSC-like traits; in addition, a Wnt-responsive CSC pathway was identified.

## Background

Laryngeal carcinoma is one of the most common head and neck malignancies, and the vast majority of cases are laryngeal squamous cell carcinoma (LSCC). At the time of initial diagnosis, approximately 60% of laryngeal cancer patients are diagnosed with clinical stage III or IV disease, which is known as advanced LSCC [1]. The 5-year overall survival (OS) rates of patients with advanced LSCC is still low due to relapse and resistance to treatment. Thus, there is a great need to understand the molecular mechanisms of LSCC tumorigenesis to identify novel prognostic molecular markers in order to develop novel therapeutic strategies.

Cancer stem cells (CSCs), a small population of cancer cells that retain stem cell-like properties [2], account for most of the development of drug resistance and tumor recurrence. CSCs have been proven to be present in various tumors, including those of lung [3], brain [4], breast [5], prostate [6], colon [7], ovarian [8], and head and neck cancer [9]. CSCs are commonly identified by surface and nonsurface biomarkers, which are conserved and specific. For most malignancies, CD133 and CD44 have been used as common biomarkers for CSCs [10–14]. Furthermore, cancer stem-like cells (CSLCs) were enriched among cancer side-population (SP) cells, so the CSCs could be regarded as CSLCs and SP cells.

Procollagen-lysine, 2-oxoglutarate 5-dioxygenase 2 (PLOD2), also known as LH2, TLH2 and BRKS2, is a membrane-bound homodimeric enzyme that specifically hydroxylates lysines in the telopeptide of procollagens [15]. This enzyme is encoded by the PLOD2 gene, which has been implicated in the extracellular matrix formation and various pathological processes. Importantly, abnormal PLOD2 expression has been observed in many types of cancer. PLOD2 was found to be overexpressed in bladder cancer [16], oral carcinoma [17], hepatocellular carcinoma [18], breast cancer [19], sarcoma [20], and renal cell carcinoma [21] and was closely related to a poor prognosis. However, PLOD2 expression has shown no differences between gastric adenocarcinoma versus normal tissues and has consistently showed no relation with prognosis in gastric cancer based on the Kaplan-Meier plotter of the datasets from Oncomine™ database. The function of PLOD2 in the collagen cross-link switch and tumor cell invasion and migration has been confirmed in relation to metastasis [15]. Therefore, PLOD2 is thought to be a novel prognostic factor in several types of cancer. However, the role of PLOD2 in laryngeal cancer is unknown, and the mechanism of PLOD2 in malignancies remains largely unexplored.

In our preliminary work, we found that PLOD2 expression was significantly increased in LSCC and was closely related to the prognosis of LSCC patients. We assumed that PLOD2 is an indicator of prognosis in LSCC patients and plays a key role in the progression of laryngeal cancer. Here, we validated this assumption in 114 LSCC patients and attempted to determine the underlying mechanism. Finally, we report a novel role of PLOD2 in enhancing CSC-like traits and conferring drug resistance in LSCC via the Wnt/β-catenin signaling pathway.

## Methods

### Patients and specimens

Specimens were collected from 114 archives of patients with LSCC who underwent surgical resection at the Department of Head and Neck Surgery of Guangdong Provincial People’s Hospital & Guangdong Academy of Medical Sciences (Guangzhou, China) from 2008 to 2015. The tumor and paired normal adjacent tissue samples were collected from each patient. All samples for qRT-PCR were obtained from the surgically resected material, immediately placed in RNAlater TissueProtect Tubes (Qiagen, Hilden, Germany) and stored at − 80 °C.Written informed consent was obtained beforehand from all patients, and the protocol was approved by the Ethical Committee of Guangdong Provincial People’s Hospital & Guangdong Academy of Medical Sciences. The median age of the patients was 62 years (range 43–85 years). There were 106 male and 8 female patients. The clinicopathological characteristics of the patients and samples are shown in Table 1. The patients were followed by telephone every 3 months during the first 2 years postoperatively and every 6 months thereafter until death.

**Table 1:** Clinicopathological characteristics of patient samples and expression of PLOD2 in laryngeal cancer

Parameters	Number of cases (%)
Gender	
Male	106 (93.0)
Female	8 (7.0)
Age (years)	
< 62	56 (49.1)
≥ 62	58 (50.9)
Clinical stage	
I	38 (33.3)
II	16 (14.0)
III	28 (24.6)
IV	32 (28.1)
T classification	
T_1_& T_2_	59 (51.8)
T_3_	29 (25.4)
T_4_	26 (22.8)
N classification	
N_0_	86 (75.4)
N_1_& N_2_& N_3_	28 (24.6)
M classification	
No	103 (90.4)
Yes	11 (9.6)
Thyroid cartilage invasion	
No	88 (77.2)
Yes	26 (22.8)
Type	
Supraglottic	35 (30.7)
Subglottic	4 (3.5)
Glottic	74 (64.9)
Transglottic	1 (0.9)
Expression of CD44	
Low expression	57 (50.0)
High expression	57 (50.0)
Expression of CD133	
Low expression	62 (54.4)
High expression	52 (45.6)
Relapse status (at follow-up)	
No	78 (68.4)
Yes	36 (31.6)
Vital status (at follow-up)	
Alive	93 (81.6)
Dead	21 (18.4)
Expression of PLOD2	
Low expression	59 (51.8)
High expression	55 (48.2)

### Immunohistochemical (IHC) staining and evaluation

Paraffin-embedded LSCC tissue sections with a thickness of 50um were prepared, and IHC staining was performed using antibodies against PLOD2, CD44 and CD133. The staining results were evaluated and scored based on both the proportion of positively stained tumor cells and the intensity of staining, and analyzed by two independent pathologists in a blinded manner. The staining index (SI) was calculated as the product of the staining intensity and the proportion of positive cells. The staining intensity score was defined as: no staining = 0, weak staining (light yellow) = 1, moderate staining (yellow brown) = 2; strong staining (brown) = 3.Cell proportions were expressed as follows: 0(no positive cells, 1(< 25% positive cells), 2(26–50% positive cells), 3(51–75% positive cells), 4(> 75% positive cells). Using this method of assessment, we evaluated protein expression by determining the SI, with possible scores of 0, 1, 2, 3, 4, 6, 8, 9, and 12. High- and low-expression samples ere defined as those with an SI ≥6 or SI < 6 respectively.

### Cell culture and stable cell line establishment

FaDu and Hep2 cell lines, the human head and neck squamous cell carcinoma lines, were selected as they are the available laryngeal cell lines in China (both from American Type Culture Collection, Manassas, VA, USA). Cells were maintained in DMEM medium (Invitrogen, Carlsbad, CA, USA) supplemented with 10% fetal bovine serum (HyClone, Logan, UT, USA) and were maintained in a humidified incubator at 37 °C with 5% CO2.

For stable cell line establishment, the indicated plasmids (pSin-EF2-puro-retro-PLOD2 and pSuper-puro-retro-PLOD2-RNAi) were packed into retrovirus in 293 T cells. Then, the viruses were harvested and FaDu and Hep2 cells were infected. After infection for 48 h, the cells were selected with medium containing puromycin (0.5 μg/ml) over 1 week.

### qRT-PCR

Total RNA was extracted from the LSCC tissue samples and cells using TRIzol reagent (Invitrogen, Carlsbad, CA, USA). qRT-PCR was performed using SYBR Green (Takara, Dalian, China) according to instruction of the manufacturer. The qRT-PCR results were analyzed, and the relative mRNA levels were calculated using the 2-ΔΔCt method. All experiments were performed at least three times in triplicate. The primer sequences used are provided in Table 2.

**Table 2:** The primer sequences were used in qRT-PCR

Gene	F	R
PLOD2	CATGGACACAGGATAATGGCTG	AGGGGTTGGTTGCTCAATAAAAA
CD44	GAAGATTTGGACAGGACAGGAC	CGTGTGTGGGTAATGAGAGGTA
CD133	TTTGGATTCATATGCCTTCTGT	CCATTGGCATTCTCTTTGAA
MMP2	TACAGGATCATTGGCTACACACC	GGTCACATCGCTCCAGACT
TCF4	CAAGCACTGCCGACTACAATA	CCAGGCTGATTCATCCCACTG
SNAIL1	TCGGAAGCCTAACTACAGCGA	AGATGAGCATTGGCAGCGAG
β-actin	CTCCATCCTGGCCTCGCTGT	GCTGTCACCTTCACCGTTCC
MYC	GGCTCCTGGCAAAAGGTCA	CTGCGTAGTTGTGCTGATGT
NANOG	TTTGTGGGCCTGAAGAAAACT	AGGGCTGTCCTGAATAAGCAG
ABCG2	CAGGTGGAGGCAAATCTTCGT	ACCCTGTTAATCCGTTCGTTTT
KLF4	CCCACATGAAGCGACTTCCC	CAGGTCCAGGAGATCGTTGAA
OCT4	CAAAGCAGAAACCCTCGTGC	TCTCACTCGGTTCTCGATACTG
GAPDH	TCCTCTGACTTCAACAGCGACAC	CACCCTGTTGCTGTAGCCAAATTC

### Western blotting

Tissue or cell lysates containing 20 μg of protein were prepared, and electrophoresed on a 10% SDS-PAGE gel under denaturing conditions, subsequently transferred to a PVDF membrane by electroblotting. The membrane was blocked in 5% nonfat milk for 60 min at room temperature, then incubated with the primary antibodies overnight at 4 °C. After washing with TBST three times, the membrane was incubated with horseradish peroxidase (HRP)-conjugated secondary antibody (BOSTER,CA,USA) for 120 min at room temperature. The antibodies used included anti-PLOD2 mouse monoclonal antibody (1:3000 dilution; mab4445, R&D); anti-CD44, anti-CD133, anti-β-catenin rabbit polyclonal antibody (1:3000 dilution; 9582, CST), anti-myc mouse monoclonal antibody (1:3000; AM926, Beyotime), anti-MDR1 rabbit polyclonal antibody (1:3000; BA1351, BOSTER), anti-MRP antibody, anti-caspase-3 mouse monoclonal antibody (1:3000; SC-7272, SANTA), α-tubulin monoclonal mouse monoclonal antibody (1:3000 dilution; T9026, Sigma-Aldrich), and anti-GAPDH antibody (1:3000 dilution; BM1623, BOSTER).

### Tumor sphere formation assay

Cells were trypsinized and seeded in six-well ultralow attachment culture plates at a density of 500 cells/well, cultured in DMEM/F12 serum-free medium (Invitrogen) with 2% B-27 Supplement (Invitrogen), 0.4% bovine serum albumin (BSA, Sigma-Aldrich), 20 ng/ml basal fibroblast growth factor (bFGF, PeproTech, Rocky Hill, NJ, USA), 20 ng/ml epidermal growth factor (EGF, PeproTech), and 5 μg/ml insulin (Sigma-Aldrich) for 11 days at 37 °C with 5% CO2. Subsequently, the numbers of spheres were counted under a microscope.

### Analysis of the SP cell fraction

Hep-2 and FaDu cells were used in the SP assay to identify stem cells that overexpressed PLOD2 and could transport Hoechst 33342 dye. The cells were suspended in RPMI 1640 medium, which containing 2% fetal bovine serum (FBS) and 2 mM DMEM, at 1 × 10^6^ cells per milliliter,then incubated at 37 °C for 20 min. Subsequently, Hoechst 33342 dye (Invitrogen) was added to a final concentration of 5 μg/mL (8.1 μM) from a 1 mg/mL stock solution, then the cells were incubated with cells for 90 min under shaking in a 37 °C bath. The control cells were incubated with 50 μM verapamil (Sigma Chemicals Co.) at 37 °C for 15 min before the addition of Hoechst 33342 dye. Hoechst 33342-labeled cells were analyzed on an FACSC alibur flow cytometer (BD Biosciences), and cell aggregates were discarded from the analysis by doublet discrimination. SP cells were visualized or sorted by red (FL8) vs blue (FL7) ultraviolet channels both in linear mode.

### Flow cytometry analysis

After transfection for 48 h, the cells were harvested by trypsinization and washed twice with cold phosphate-buffered saline (PBS). Then, the cells were resuspended in binding buffer and stained with 3.5 μL of Annexin V-FITC and 5 μL of propidium iodide (PI) for 15 min at room temperature in the dark, according to the manufacturer’s instructions. Apoptotic cells were observed using a FACSCalibur flow cytometer system (BD Biosciences, San Jose, CA, USA) and analyzed using Summit5.2 software (Beckman Coulter, Indianapolis, IN). Flow cytometry analysis of Hoechst 33342 staining was performed on cells stably expressing PLOD2 or pBabe (vector) (upper panel). FTC (5 μM) was added before Hoechst 33342 staining (lower panel). The proportion of cells in the SP fraction was then presented.

### Tumor xenografts

All animal experiments were approved by the Ethical Committee of Guangdong Provincial People’s Hospital & Guangdong Academy of Medical Sciences. BALB/c-nu mice (5–6 weeks old, Guangdong Animal Laboratory, Guangzhou, China) were used in this study. The mice were maintained under pathogen-free conditions according to the institutional guidelines for animal welfare. The mice were injected subcutaneously with 1 × 10^5^cells. Forty mice were randomly divided into four groups and injected with equal numbers of cells, and tumor formation was monitored for up to 10 days. Every group was allocated into 2 subgroups. Then one subgroup were treated with cisplatin (5 mg/kg, intraperitoneal injection, once a week) for another 20 days and the other subgroup as control. The length and width of tumors were measured with calipers, and the volumes were calculated using the equation (length× width2)/2. After the mice were euthanized, the tumors were excised and used to pathological examination.

### TOP/FOP-flash assay

Hep-2 and FaDu cells were plated in 24-well plates. Then cells were transiently transfected with plasmids containing firefly luciferase reporters and recombinant promoter reporter constructs. Luciferase and renilla signals was measured after incubation for 48 h by using the Dual Luciferase Assay kit (Promega, Madison, WI). Each experiment was performed at least three times in triplicate according to the manufacturer’s instructions.

### Statistical analysis

The SPSS statistical software package (version 20.0) was used for all statistical calculations. The difference in PLOD2 expression between the LSCC and the paired normal adjacent tissues and the correlations between the protein expression levels and clinicopathological variables were analyzed using the Chi-square test or Fisher’s exact test. The differences among the groups were compared by one-way ANOVA, and the data are expressed as the mean ± SD. The statistical differences were set at *P* < 0.05.

## Results

### PLOD2 overexpression is correlated with progression and a poor prognosis in LSCC patients

**Fig. 1:**
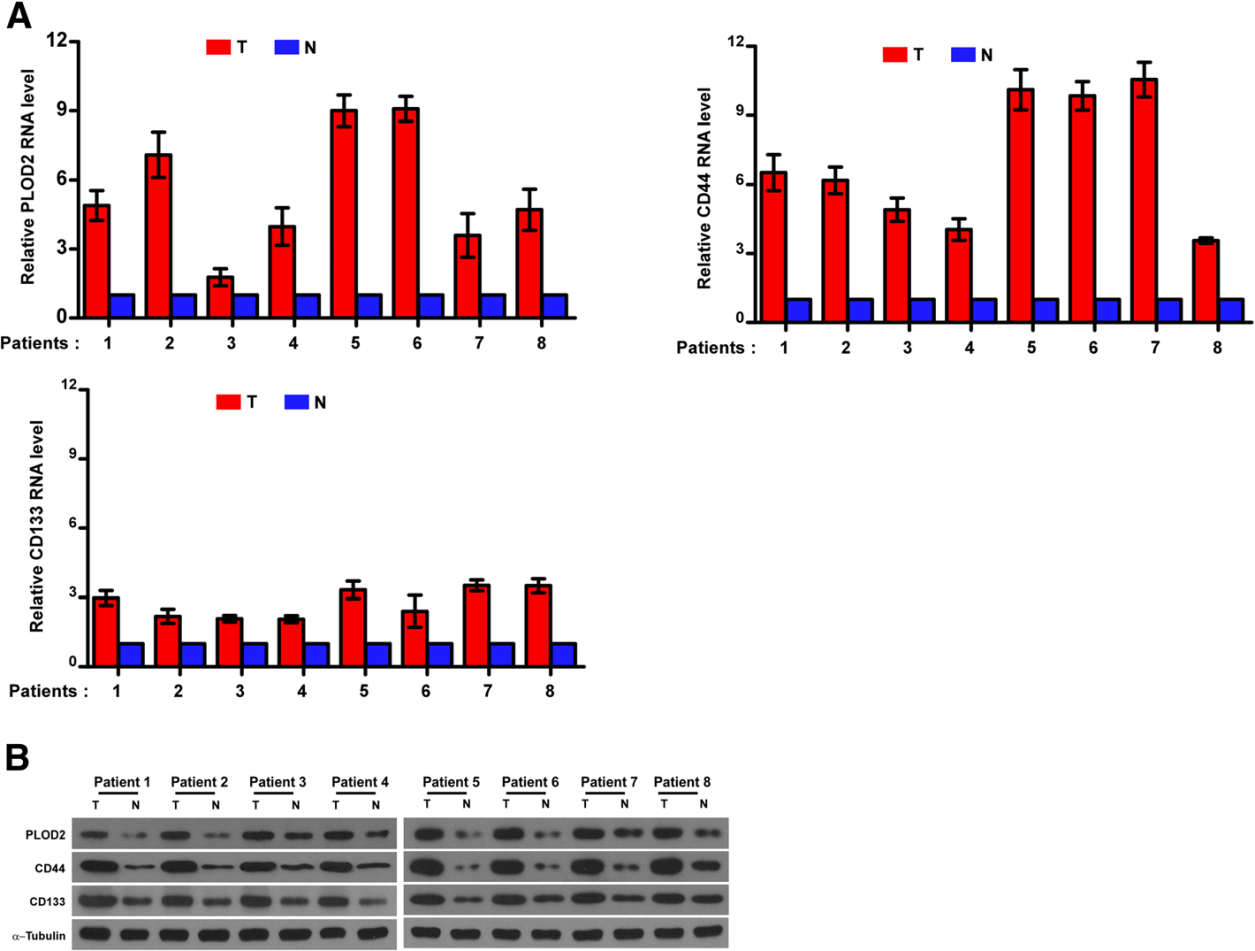
PLOD2, CD44, and CD133 mRNA expression levels and protein in 8 pairs of human LSCC tissues and matched adjacent nontumor tissues. **a** Relative PLOD2, CD44, and CD133 RNA expression in 8 pairs of LSCC tumor tissues (T) and matched adjacent nontumor tissues (N). **b** Relative PLOD2, CD44, and CD133 protein expression in human LSCC tumor tissues and matched adjacent nontumor tissues. α-Tubulin was measured as the loading control

**Fig. 2:**
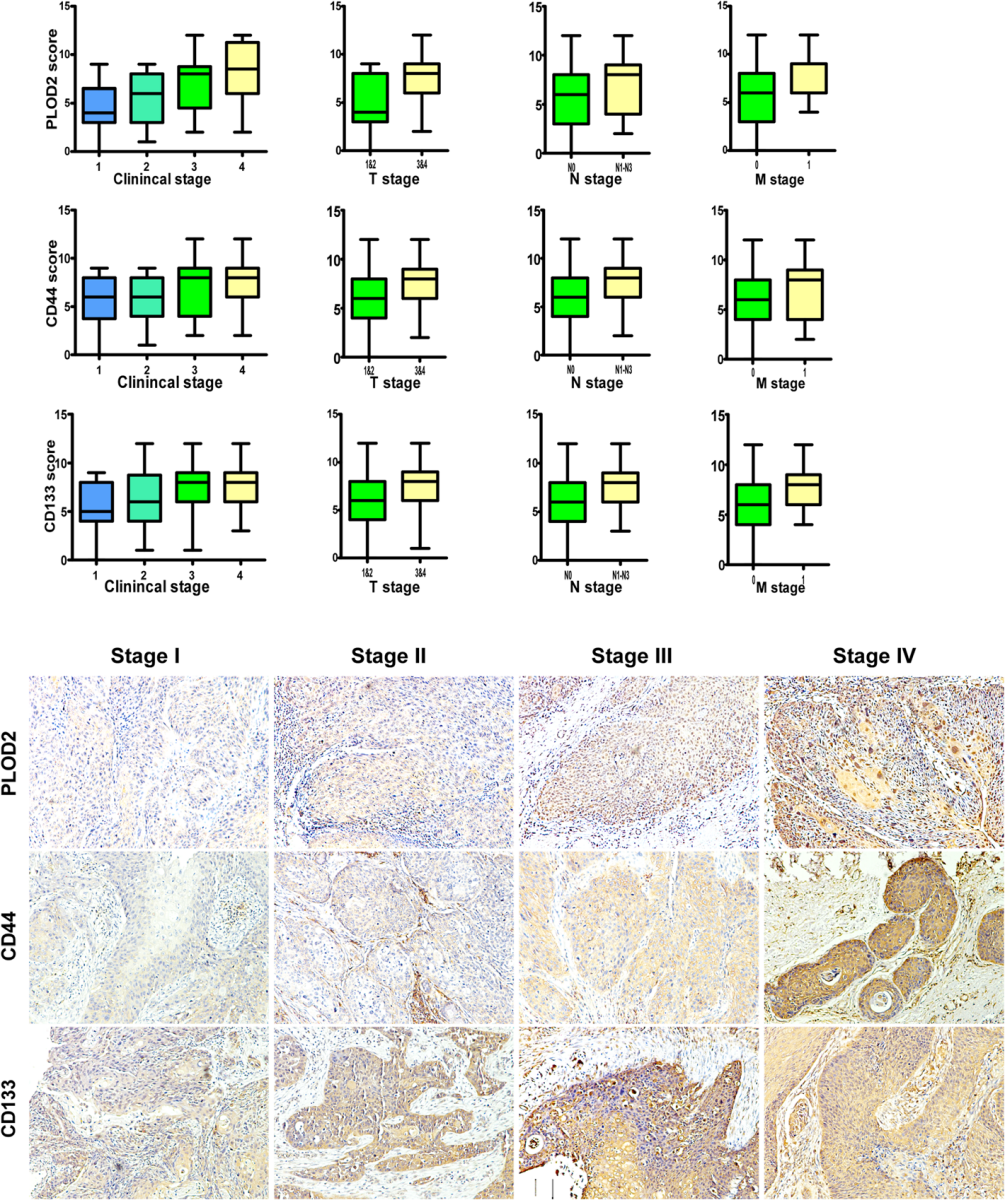
IHC analysis of the expression levels of PLOD2, CD44, and CD133 in laryngeal tumor samples with different clinical stages (1, 2, 3 and 4), T stages (T1 & T2, T3 & T4), N stages (N0, N1-N3), and M stages (M0, M1). The expression of PLOD2, CD44 and CD133 was increased in the advanced-stage LSCC tumor tissues. Magnification: × 200

We found that PLOD2 was highly expressed in head and neck squamous carcinoma in the publicly available human cancer datasets of The Cancer Genome Atlas (TCGA). To determine the difference of PLOD2 expression in LSCC, we performed qRT-PCR and western blot analyses in 8 pairs of human laryngeal cancer tissues and the adjacent normal laryngeal tissues, and revealed that PLOD2 was upregulated at both the protein and mRNA levels in laryngeal cancer tissues (Fig. 1), suggesting that PLOD2 is upregulated in human laryngeal cancer. Consistently, we validated not only that the level of PLOD2 protein expression was upregulated in the LSCC tissues compared with that in the paired adjacent normal tissues by the IHC staining of 114 archived LSCC tissues but also that PLOD2 expression increased along with tumor progression. Furthermore, we found similar trends in the expression of CD44/CD133 (Fig. 2) accompanied with PLOD2 in tissues. CD44 and CD133 have been widely used as markers to identify CSCs.

The clinical significance of the high PLOD2 expression was further assessed (Table 1). According to the SI, PLOD2 expression was divided into the following two levels: high expression (PLOD2 high, *n* = 55) and low expression (PLOD2 low, *n* = 59). We found that PLOD2 expression was positively correlated with the clinical, T, N, and M stage and the expression of CD44/CD133 (*P* < 0.05) (Fig. 2, Table 3).

**Table 3:** Correlation between PLOD2 expression and clinicopathological characteristics of laryngeal cancer patients

Characteristics	PLOD2		χ^2^ test *p*	Fisher’s exact test *p*
	Low, no. cases (%)	High, no. cases (%)		
Clinical stage				
I&II	38 (64.4)	16 (29.1)	< 0.001	< 0.001
III&IV	21 (35.6)	39 (70.9)		
T classification				
T1& T2	42 (71.2)	17 (30.9)	< 0.001	< 0.001
T3& T4	17 (28.8)	38 (69.1)		
N classification				
N_0_	49 (83.1)	37 (67.3)	0.051	0.080
N_1_& N_2_& N_3_	10 (16.9)	18 (32.7)		
M classification				
No	56 (94.9)	47 (85.5)	0.087	0.116
Yes	3 (5.1)	8 (14.5)		
Expression of CD44				
Low expression	50 (84.7)	7 (12.7)	< 0.001	< 0.001
High expression	9 (15.3)	48 (87.3)		
Expression of CD133				
Low expression	46 (78.0)	16 (29.1)	< 0.001	< 0.001
High expression	13 (22.0)	39 (70.9)		
Age (years)				
< 62	30 (50.8)	26 (47.3)	0.703	0.712
≥ 62	29 (49.2)	29 (52.7)		

We further analyzed the correlations between the clinicopathological features and prognosis of the LSCC patients. All the variables were entered stepwise into the multivariable Cox proportional hazard model by the forward conditional method. M stage (HR = 3.463; 95% CI 1.087–11.031; *P* = 0.036), thyroid cartilage invasion (HR = 3.180; 95% CI 1.136–8.898; *P* = 0.028) and high PLOD2 expression (HR = 4.742, 95% CI 1.452–15.485; *P* = 0.010) were independent prognostic factors of OS in the patients with LSCC (Table 4), which is consistent with the results of the univariable analysis.

**Table 4:** Univariate and multivariate analysis of factors associated with overall survival in 114 laryngeal cancer patients

Characteristics	Univariate analysis		Multivariate analysis	
	HR (95% CI)	*P* values	HR (95% CI)	*P* values
Age	0.928 (0.386–2.233)	0.868	2.440	0.118
Gender	1.857 (0.248–13.910)	0.547	2.856	0.091
T stage	8.280 (2.408–28.478)	0.001*	5.624 (1.594–19.840)	0.007*
N stage	2.229 (0.910–5.461)	0.080	0.081	0.776
M stage	3.935 (1.418–10.917)	0.009*	2.744 (0.987–7.629)	0.053
Clinical stage	6.252 (1.827–21.396)	0.004*	0.398	0.528
Relapse	3.377 (1.395–8.175)	0.007*	2.153	0.142
Thyroid cartilage invasion	4.005 (1.634–9.819)	0.002*	0.699	0.403
CD44	5.845 (1.936–17.647)	0.002*	1.459	0.227
CD133	3.769 (1.436–9.891)	0.007*	0.626	0.429
PLOD2	6.720 (2.193–20.588)	0.001*	4.559 (1.356–15.325)	0.014*

*HR* hazard ratio, *CI* confidence interval

* *p* < 0.05

**Fig. 3:**
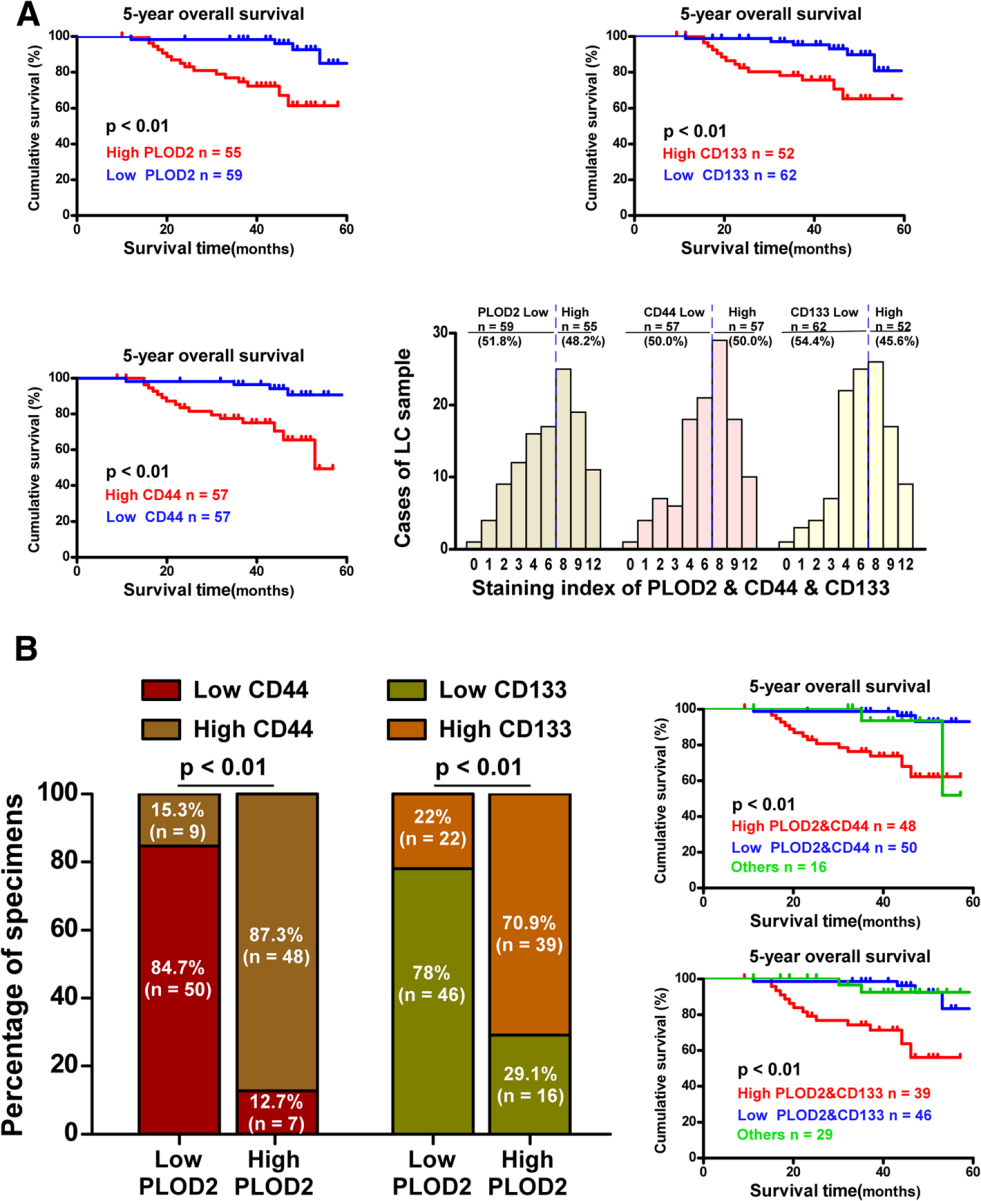
OS curves based on different PLOD2 expression levels. **a** Kaplan-Meier survival curves for LSCC patients with high PLOD2, CD44 and CD133 expression (red line) versus low PLOD2, CD44 and CD133 expression (blue line). **b** Kaplan-Meier survival curves for LSCC patients with high PLOD2 and CD44/CD133 expression (red line) versus low PLOD2 and CD44/CD133 expression (blue line)

Furthermore, Kaplan-Meier plotter revealed that patients with high PLOD2 expression had shorter 5-year OS (*P* = 0.000), and the same correlation was found between CD44/CD133 expression and 5-year OS (Fig. 3a). Intriguingly, CD44/CD133 expression was high in the majority of the tissues with high PLOD2 expression, and vice versa (Fig. 3b).

These results reveal that PLOD2 overexpression might promote LSCC progression, leading to poor clinical outcomes. Due to the correlation between PLOD2 and CD44/CD133 expression, PLOD2 might contribute to increasing cancer cell-like characteristics in LSCC.

### PLOD2 contributes to the CSC-like properties of Hep-2 and FaDu cells

**Fig. 4:**
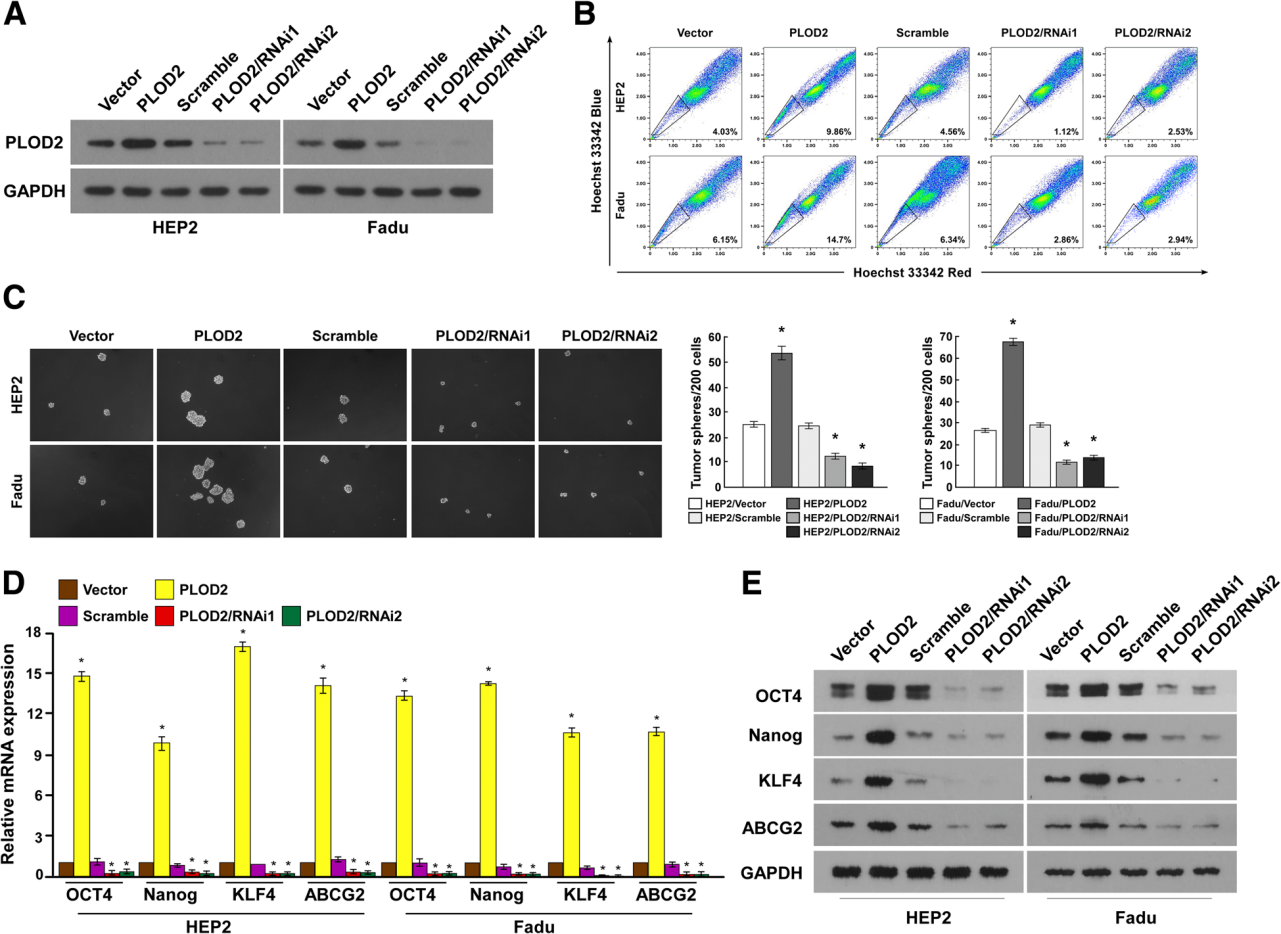
Manifestations of PLOD2 maintaining CSC characteristics. **a** Hep-2 and FaDu cell lines were engineered for the overexpression or silencing of PLOD2. **b** The SP was analyzed in the different cell lines by multiparametric flow cytometry. **c** Hep-2 and FaDu cell spheres cultured in medium were photographed; representative images are shown. Scale bar = 50 μm. **d** The mRNA expression levels of genes representing CSC markers were upregulated. **e** The protein expression levels of genes representing CSC markers were upregulated. *P < 0.05

Stably infected Hep2 and FaDu cells were established with PLOD2 overexpressed and silenced (Fig. 4a). SP cells could efflux dye and fell to the “side” of the bulk of the positively stained cells in the FACS analysis plots [22]. It has been confirmed that SP cells have stem cell-like characteristics [23]. In the study, SP cells were used to valid the CSCs characteristic. The flow cytometry assays revealed smaller SP^+^ subpopulations among the PLOD2 siRNA-treated Hep-2 and FaDu cells compared than the controls (Fig. 4b).

Moreover, we found that silencing PLOD2 strongly inhibited Hep-2 and FaDu tumor sphere formation, generating approximately 2-fold fewer spheres with an approximately 2-fold lower cell content compared with that in the control cells (Fig. 4c). These results suggest that PLOD2 is essential for the maintenance of LSCC stem cell properties and inhibiting apoptosis. Subsequently, we analyzed the expression of classic embryonic stem cell transcription factors, including OCT4, Nanog, KLF4 and ABCG2 at the mRNA and protein levels (Fig. 4d, e). These four genes were upregulated in the PLOD2-overexpressing cells. Hence, PLOD2 may activate the above genes to increase CSC-like characteristics via a certain signaling pathway.

### Upregulation of PLOD2 confers drug resistance in LSCC in vitro and in vivo

**Fig. 5:**
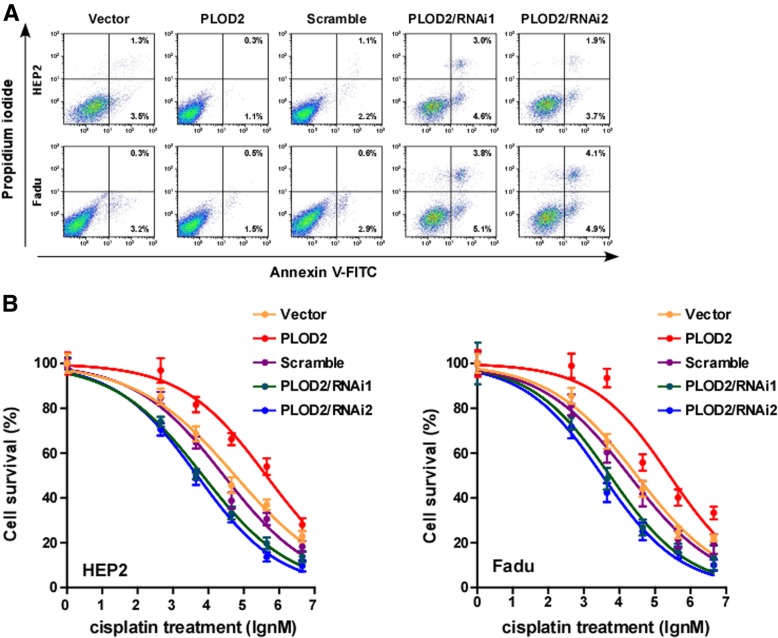
PLOD2 could promote drug resistance in laryngeal cancer cells. **a** Apoptotic scatter plot; Annexin V-FITC and PI staining of the indicated cells treated with DDP (50 μM) for 48 h. **b** IC50 of DDP in the indicated cells. Each bar represents the mean ± SD of five independent experiments. *P < 0.05. FITC, fluorescein isothiocyanate; PI, propidium iodide

Few studies have examined the association between PLOD2 and drug resistance, and abnormal regulation of apoptosis attributes for most of drug resistance. To investigate the anti-apoptotic role of PLOD2 in LSCC progression, Hep-2 and FaDu cells stably expressing PLOD2 were established (Fig. 3a). The percentage of apoptotic cells among PLOD2-overexpressing LSCC cells treated with a drug (cisplatin, DDP) was much lower than that among the control cells but much higher than that among cells with silenced PLOD2 expression (Fig. 5a). Moreover, the IC50 value of the drug (DDP) was significantly decreased in the PLOD2-overexpressing LSCC cells but increased in the cells with silenced PLOD2 expression (Fig. 5b), suggesting that PLOD2 enhanced the resistance of LSCC cells to DDP in vitro.

**Fig. 6:**
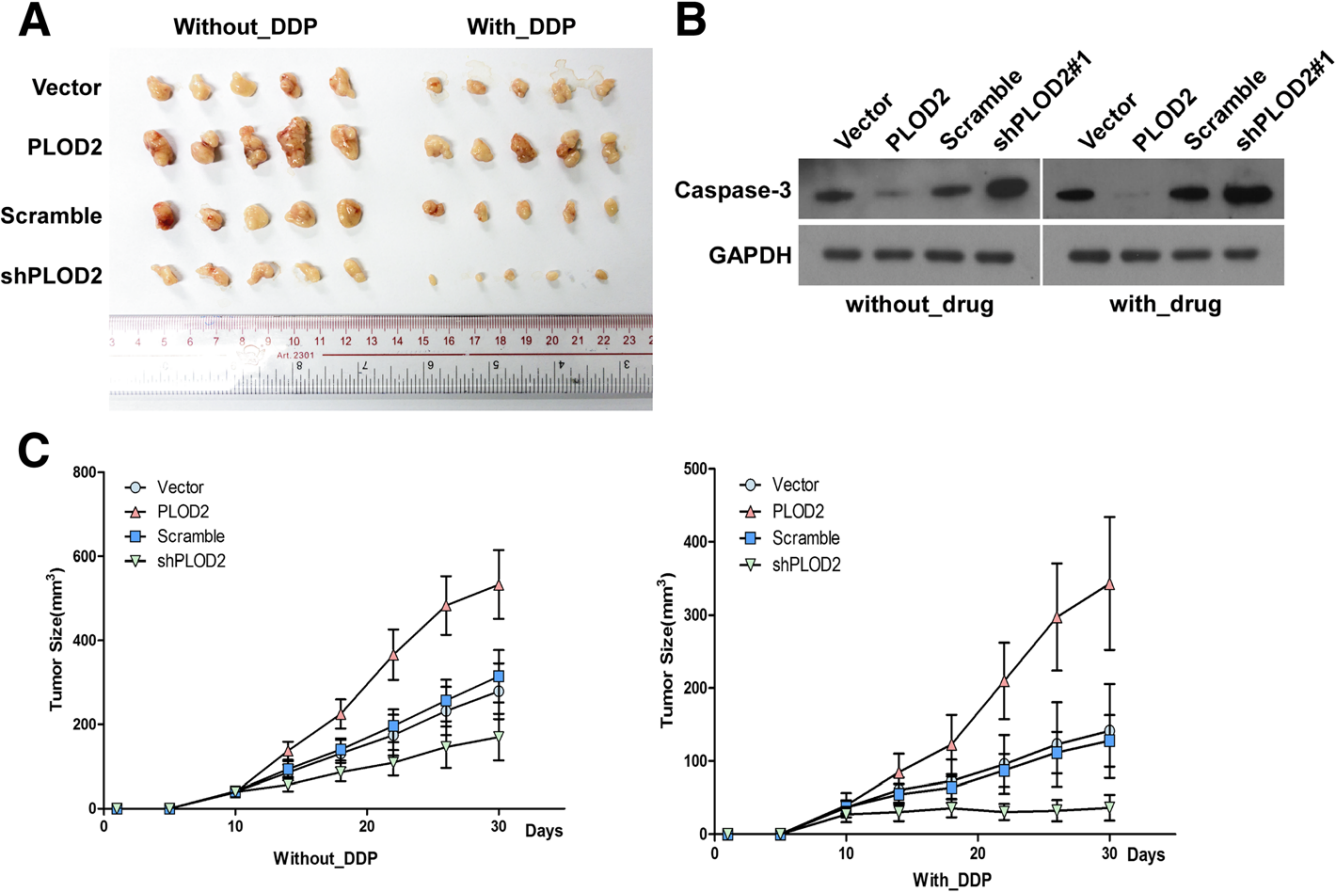
PLOD2 overexpression contributes to drug resistance in Hep-2 cells in vivo. **a** Representative photographs of tumors isolated from the experimental mice (with/without DDP treatment). **b** Cleaved caspase 3 was significantly decreased in PLOD2-overexpressing Hep-2 cells. **c** Tumor volumes of the experimental mice during the treatment period. Each bar represents the mean ± SD of five independent experiments

To validate the contribution of PLOD2 of laryngeal cancer to chemoresistance, we used nude mice model. The nude mice were divided into four groups and subcutaneously inoculated with Hep-2 cells transfected with vector, PLOD2, scrambled, and PLOD2 shRNA (shPLOD2) respectively. Then, the mice were treated with DDP once a week after 10 days. As shown in Fig. 6a and c, treatment with shPLOD2 plus DDP resulted in a significant reduction in tumor growth; however, compared with the control, PLOD2 overexpression resulted in a significant increase in tumor growth. Interestingly, the protein level of cleaved caspase-3 was significantly decreased in PLOD2-overexpressing Hep-2 cells (Fig. 6b).

### Upregulation of PLOD2 activates the Wnt signaling pathway in LSCC cells

**Fig. 7:**
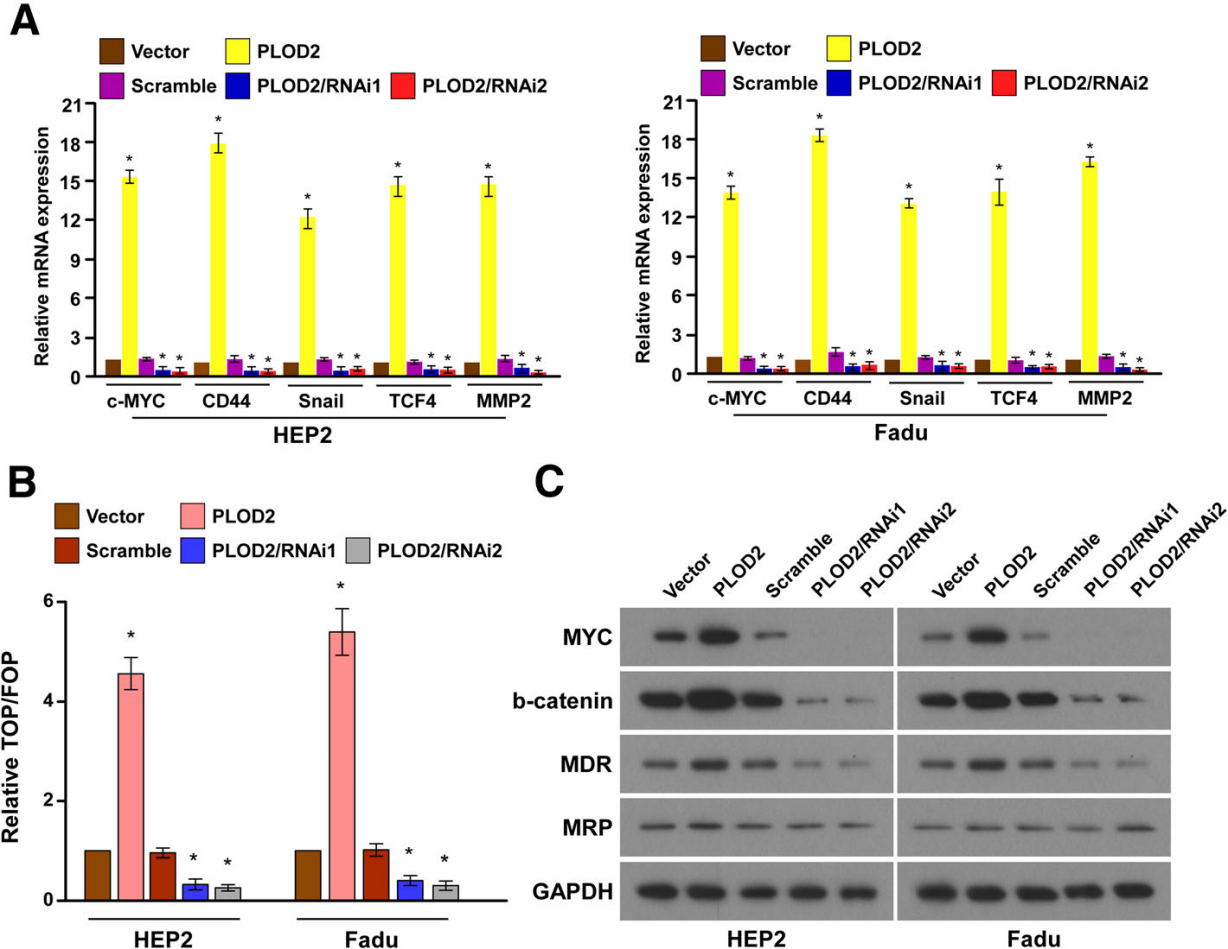
PLOD2 overexpression could activate the Wnt signaling pathway. **a** The mRNA levels of downstream genes of the Wnt signaling pathway(c-myc, CD44, Snail, TCF4, and MMP-2) were upregulated by PLOD2. β-Actin was used as an internal control. **b** Dual luciferase assay showing the effect on TOP/FOP reporter activity in LSCC cells following the overexpression and silencing of PLOD2. **c** Immunoblot assay of c-myc, β-catenin, MDR-1, and MRP protein levels in LSCC cells transfected with PLOD2 and controls. GAPDH was used as an internal control

By analyzing the PLOD2 mRNA expression levels and Wnt-regulated gene signatures in laryngeal cancer cells, we found that PLOD2 overexpression upregulated the downstream genes of the Wnt signaling pathway (Fig. 7a). Thus, TOP-Flash and FOP-Flash reporters co-transfected with PLOD2 overexpression were constructed to determine whether PLOD2 modulates the canonical Wnt/β-catenin signaling pathway. As expected, the overexpression of PLOD2 significantly increased the activity of TOP/FOP, whereas the silencing of PLOD2 significantly reduced the reporter activity (Fig. 7b). Moreover, western blotting revealed that the levels of c-myc and β-catenin, which are downstream genes of the Wnt signaling pathway, were dramatically upregulated in the PLOD2-overexpressing cells but downregulated in the cells with silenced PLOD2 expression (Fig. 7c). As shown by western blotting results, the expression of the drug resistance-related genes MDR-1 and MRP was significantly elevated in the PLOD2-overexpressing cells (Fig. 7c).

## Discussion

Despite many advances in diagnostic methods and treatments, patients with advanced LSCC experience tend to relapse and metastasis and have a poor prognosis. Previous studies have suggested that PLOD2 is overexpressed in sarcoma [20], bladder cancer [16], renal cell carcinoma [21], glioblastoma [24], cervical cancer [25], oral carcinoma [17], bone metastasis [26] and other types of cancers [15]. The overexpression of PLOD2 is closely related to a poor prognosis in lung cancer and breast cancer, based on the Kaplan-Meier analysis. In our study, PLOD2 was overexpressed in LSCC tissues, and the LSCC patients with PLOD2 overexpression presented a lower OS rate; additionally, the patients with advanced-stage LSCC had higher PLOD2 expression. Hence, consistent with previous research on other malignancies, we showed that PLOD2 might be a biomarker of a poor prognosis in LSCC.

To determine the role of PLOD2 in the progression of LSCC, Hep-2 and FaDu cell lines were engineered to validate the function of PLOD2 in vitro. The flow cytometry results show smaller SP^+^ subpopulations among Hep-2 and FaDu cells with silenced PLOD2 expression than among the controls. In addition, PLOD2 expression maintained the CSC-like properties of Hep-2 and FaDu cells in vitro. Moreover, the levels of CSC surface markers were increased in the PLOD2-overexpressing cells. To date, there has been little research on correlations between PLOD2 and CSCs. In 1996, PLOD2 was first reported in breast cancer research by Smith [15]. Recently, fundamental evidence suggests that PLOD2 plays critical roles in the progression of cancer. The function and mechanism of PLOD2 in several types of cancer have been explored. The published reports suggest that PLOD2 plays a key role in collagen cross-linking, promoting tumor cell invasion and migration [27]. As functional studies have shown, PLOD2 hydroxylates telopeptidyl lysine residues on collagen, lowers levels of lysine aldehyde-derived cross-links, increases tumor stiffness, and enhances tumor cell invasion and metastasis [28]. In this study, we found that the expression of CD44 and CD133 increased as PLOD2 expression increased at both the mRNA and protein levels. In addition, some other markers of stem-like cells, such as ABCG2, KLF4, OCT4, and Nanog, co-expressed with PLOD2 were found to be highly expressed. In vitro, we also found that PLOD2 overexpression in Hep-2 and FaDu cells strongly promoted tumor sphere formation. Meanwhile, flow cytometry analysis revealed smaller SP^+^ subpopulations among Hep-2 and FaDu cells with silenced PLOD2 expression compared with those among the controls. Altogether, these data suggest that PLOD2 could enhance the CSC-like phenotype of laryngeal cancer cells and that PLOD2 plays a novel role in malignancies.

CSCs are responsible for tumor relapse, metastasis and therapy resistance. Numerous studies emerged focusing on the contribution of CSCs to the development of multidrug resistance (MDR) in many types of cancers. In this study, we found that PLOD2 overexpression conferred drug resistance in LSCC in vitro and in vivo. Thus, PLOD2 may contribute to drug resistance in LSCC by maintaining CSC-like characteristics.

By the abnormal activation of signaling pathways, such as the Notch, Hedgehog, NF-κB, Wnt, and PI3K-AKT signaling pathways [13],CSCs maintain an undifferentiated state. Gene set enrichment analysis (GSEA) was used to predict the activation of a given pathway due to the overexpression of PLOD2. The canonical Wnt signal pathway was found to be significantly related to high PLOD2 expression. It is known that Wnt/β-catenin signaling is activated by oncogenic factors or extracellular cues and drives the CSC phenotype and promote uncontrolled cell growth and malignant progression [29–31]. Indeed, overactivation of the canonical Wnt pathway has been found in many types of tumors. Notably, head and neck squamous cell carcinoma has been shown to correlate with high Wnt/β-catenin activity to promote epigenetic changes associated with an open chromatin structure and the induction of stem cell gene signatures [32]. Our data show that downstream target genes of Wnt pathway, such as c-myc and β-catenin, were activated in PLOD2-overexpressing LSCC cells. When PLOD2 was overexpressed, the activity of the TOP/FOP reporter was also significantly increased. Hence, PLOD2 may increase the CSC-like properties of cells in laryngeal cancer by activating the Wnt/β-catenin signaling pathway, which is supported by previous reports. Meanwhile, the drug-resistance-related genes MDR-1 and MRP were activated by PLOD2.

CD44 and CD133 are used to identify the CSCs in solid tumors [33]. CD 133 has been suggested to perform several functions, and approximately 16 alternative splicing patterns of CD133 are generated by five alternative promoters in many tissues [34]. Therefore, there is a great need to explore the crosstalk between PLOD2 and CD133. CD44 is a target of the Wnt pathway [35], and overexpression of PLOD2 could promote the expression of downstream genes of Wnt/β-catenin signaling. Hence, we infer that PLOD2 may activate the Wnt pathway, leading to the upregulation of CD44 .

Recently, mounting studies have provided evidence that Wnt/β-catenin signaling plays a key role in CSCs by modulating epigenetic changes that define distinct chromatin states [36]. Here, we infer that PLOD2 may promote the translation or activity of Wnt/β-catenin to maintain CSCs in LSCC and thus contribute to cancer cell drug resistance. Further studies are needed to verify our inferences.

## Conclusions

We provide new insights into the role of PLOD2 in LSCC progression. Our research indicates that PLOD2 is a prognostic indicator in LSCC and confers drug resistance in LSCC by increasing CSC-like properties through activating the canonical Wnt pathway. Further research focus on the underlying mechanism should be performed.

## Data Availability

The datasets used and/or analyze in the current study are available from the corresponding author upon reasonable request.

## Abbreviations

CI: Confidence interval
CSC: Cancer stem cell
CSCL: Cancer stem cell-like
DDP: Cisplatin
HR: Hazard ratio
IHC: Immunohistochemistry
LSCC: Laryngeal squamous cell cancer
MDR: multidrug resistance
PLOD2: Procollagen-lysine, 2-oxoglutarate 5-dioxygenase 2
SD: Standard deviation
SI: Staining index
